# Correlation of Epicardial Fat Thickness With the Severity of Coronary Artery Disease

**DOI:** 10.7759/cureus.99005

**Published:** 2025-12-11

**Authors:** Kadappa B Nagoni, Rajesh Nandal, Manu Mathew, Bimal K Agrawal, Varun Bhutani

**Affiliations:** 1 Internal Medicine, Maharishi Markandeshwar (MM) Institute of Medical Sciences and Research, Ambala, IND; 2 Cardiology, Maharishi Markandeshwar (MM) Institute of Medical Sciences and Research, Ambala, IND

**Keywords:** coronary angiography, coronary artery disease, echocardiography, epicardial adipose tissue, gensini score

## Abstract

Objective: This study aimed to assess the correlation between epicardial fat thickness (EFT) and the severity of coronary artery disease (CAD).

Methods: In this cross-sectional observational study, conducted at a tertiary care hospital in India, echocardiographic EFT measurements were correlated with CAD severity using the Gensini score from coronary angiography in fifty patients. Participants included were those undergoing percutaneous coronary angiography with suspected CAD, aged above 18 and without significant comorbidities.

Results: The study found a significant correlation between increased EFT and the severity of CAD. The mean EFT was 5.24 mm in CAD patients compared to 2.94 mm in non-CAD patients (p < 0.001). EFT was significantly higher in patients with triple vessel disease (6.25 mm) compared to those with normal angiography results (2.94 mm) (p < 0.001). Additionally, EFT positively correlated with the Gensini score (Pearson correlation coefficient: 0.697, p < 0.001). Among the risk factors, diabetes showed a significant association with increased EFT.

Conclusion: EFT serves as a promising non-invasive biomarker for CAD severity. The strong association between EFT and CAD severity, especially in males and older adults, emphasizes the importance of targeted screening and preventive strategies. Future studies should validate these findings to support EFT integration into clinical practice for early CAD risk assessment.

## Introduction

Coronary artery disease (CAD) is a condition characterized by insufficient blood and oxygen supply to the myocardium due to the narrowing or blockage of coronary arteries. CAD represents a significant portion of global health concerns, accounting for approximately 2.2% of the overall disease burden and 32.7% of cardiovascular diseases (CVDs) worldwide [1].

Within the heart, two main types of fats are present: pericardial and epicardial fat. Epicardial fat serves as a major energy source for the heart's cells, known as cardiomyocytes, which primarily rely on oxidation of fats for the production of energy [2].

Recent studies indicate that increased thickness of epicardial fat is linked with a greater risk of cardiovascular disease. Epicardial fat thickness (EFT) assessment using echocardiography has proven to be a useful and non-invasive method for understanding its role in cardiovascular health. Epicardial fat thickness measurement provides valuable information for assessing cardiovascular health and identifying individuals at higher risk of CAD [3].

## Materials and methods

This observational cross-sectional study was done in the inpatient departments of cardiology and internal medicine at a tertiary care hospital in India over the course of one year. The goal was to establish a relation between epicardial fat thickness determined by echocardiography and degree of CAD assessed using the Gensini score from coronary angiography. 

Fifty symptomatic patients undergoing percutaneous coronary angiography (PTCA) for suspected CAD were included. Inclusion criteria required participants to be over 18, while exclusions were applied to those with significant comorbidities or previous interventions.

Subjects were categorized into two groups: CAD and non-CAD. Patients who had normal coronary angiograms or insignificant stenosis were considered as non-CAD. Significant stenosis was taken as ≥50% narrowing in the left main coronary artery and ≥70% narrowing in major epicardial arteries [4]. CAD patients were divided into three categories: single, double, and triple vascular disease (SVD, DVD, and TVD). The CAD severity was measured using the Gensini score [5].

Epicardial fat thickness assessment on echocardiography

**Figure 1:**
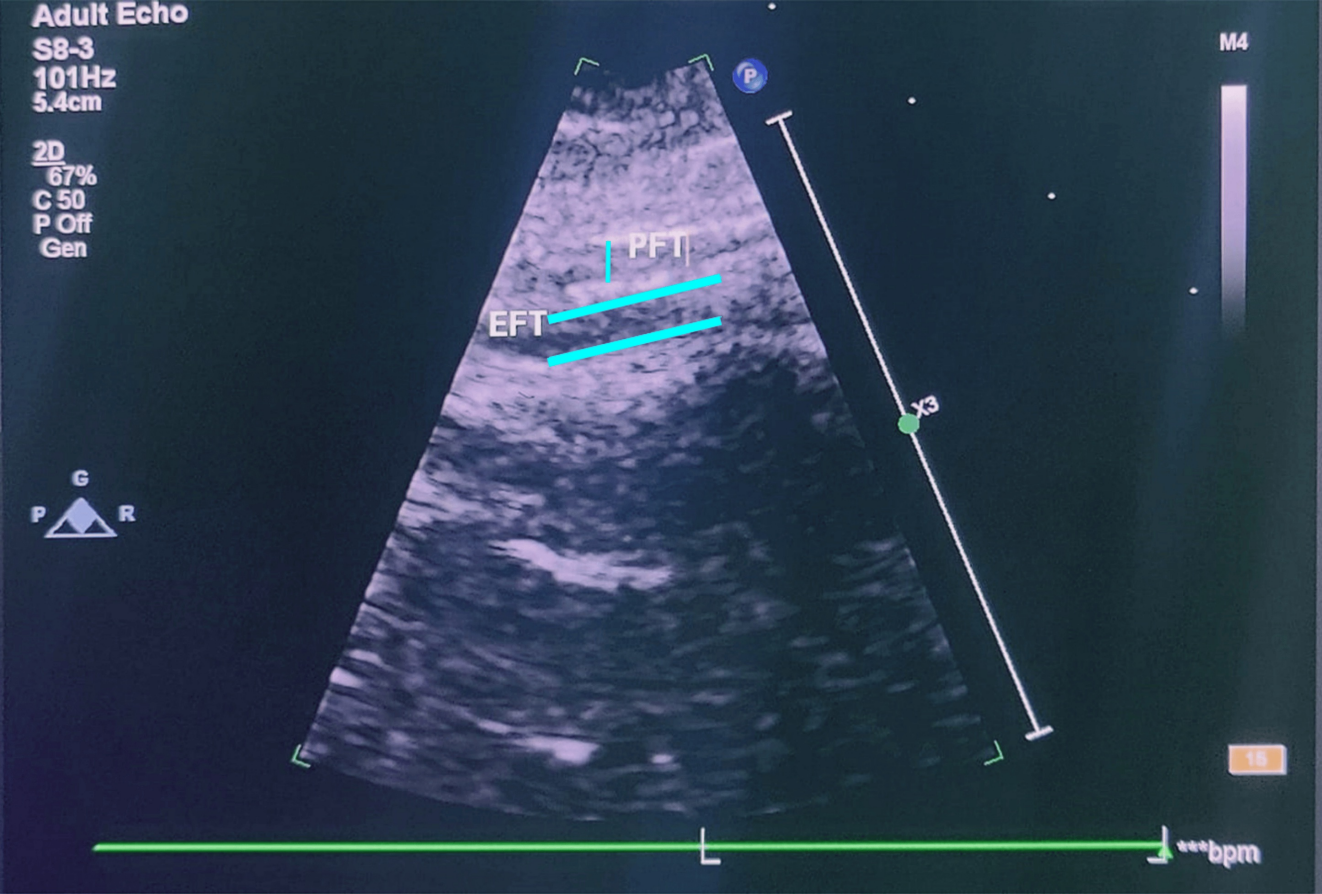
Epicardial fat thickness assessment on echocardiography. EFT: epicardial fat thickness; PFT: pericardial fat thickness.

EFT was calculated using parasternal long-axis views of the right ventricular free wall during the end of diastole. Measurements were done perpendicular to the right ventricle's free wall. Measurements from various observers were calibrated using the aortic annulus as an anatomical reference point. For statistical analysis, the mean value from three cardiac cycles was utilized [6] (Figure 1).

## Results

Patients with CAD had an average EFT of 5.24 millimeters with a standard deviation of 1.58 millimeters (5.24±1.58), whereas those without CAD had an average of 2.94 millimeters with a standard deviation of 0.89 mm (2.94±0.89). This indicates that the thickness of epicardial fat is considerably greater in CAD patients than in patients without CAD, suggesting a potential link between increased epicardial fat and the presence of CAD.

The mean EFT for males was 4.73 mm (SD = 1.74), whereas it was 4.05 mm (SD = 1.76) for females. The t-test value was 1.94 with a p-value of 0.05, suggesting that the difference in EFT between genders is statistically significant. This suggests that males have higher epicardial fat thickness compared to females, which may explain the observed higher percentage of CAD in males.

Our findings show that hypertension did not significantly affect epicardial fat thickness. The presence of diabetes was significantly associated with increased EFT. Dyslipidemia, alcohol use, and smoking did not significantly impact EFT (Table 1).

**Table 1:** Mean EFT correlation with risk factors of CAD. EFT: epicardial fat thickness; CAD: coronary artery disease.

Risk factors	Yes, mean EFT±SD (in mm)	No, mean EFT±SD (in mm)	T-test	P-value
Hypertension	4.42±1.71	4.5±1.83	0.23	0.82
Diabetes	4.91±1.86	4.03±1.58	2.55	0.01
Dyslipidemia	4.29±1.94	4.29±1.69	0.00	1
Smoking	4.7±1.84	4.3±1.73	1.12	0.27
Alcohol	4.93±1.73	4.28±1.77	1.19	0.25

The mean epicardial fat thickness increased progressively from normal (2.94 mm, SD = 0.89) to SVD (4.3 mm, SD = 1.26), DVD (5 mm, SD = 1.26), and TVD (6.25 mm, SD = 1.65). The ANOVA test results show an F-value of 34.24 with a greatly significant p-value of 0.001, indicating a strong statistical difference in epicardial fat thickness among the various groups. This suggests a significant correlation between increased EFT and the severity of CAD as diagnosed by angiography, with thicker epicardial fat being associated with more severe disease (Table 2).

**Table 2:** Mean epicardial fat thickness correlation with coronary angiography findings. SVD: single vascular disease; DVD: double vascular disease; TVD: triple vascular disease; EFT: epicardial fat thickness.

Angiography	Mean EFT	SD	ANOVA F-statistic	P-value
Normal	2.94	0.89	34.24	0.001
SVD	4.3	1.26		
DVD	5	1.26		
TVD	6.25	1.65		

**Figure 2:**
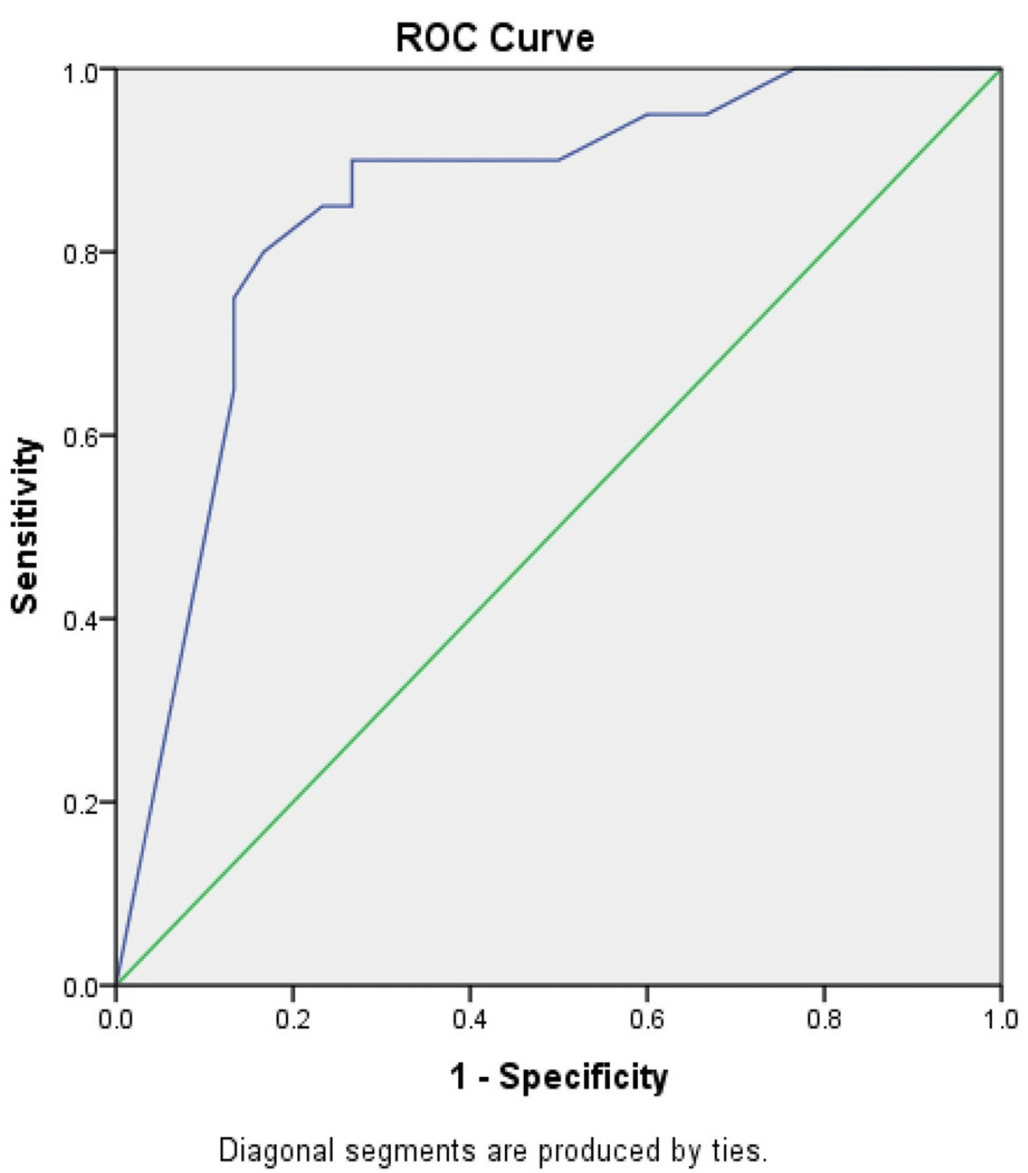
ROC curve. ROC: receiver operating characteristic.

Our findings showed a robust positive correlation between EFT and the Gensini score, with Pearson and Spearman coefficients of 0.697 and 0.673, respectively, both being statistically significant. The receiver operating characteristic (ROC) curve for EFT predicted the severity of CAD with a sensitivity of 78.79% and specificity of 76.47%, with the curve located in the upper-left corner. Excellent performance, as indicated by the area under the curve, validates EFT as a useful non-invasive biomarker in assessing CAD severity (Figure 2).

## Discussion

Coronary artery disease is a prevalent health problem with significant patient symptomatology, morbidity, and mortality. It is the major single cause of death and disability adjusted life years lost worldwide [7]. Approximately 80% of the surface of the heart is made up of white adipose tissue, known as epicardial fat, which accounts for about 20% of heart's total weight and is the main energy resource for the heart's cells, which depends primarily on fatty acid oxidation for energy [8].

Current studies indicate that increased thickness of epicardial fat is associated with a higher incidence of cardiovascular disease. Two main theories link EFT to cardiovascular disease risk: (1) its association with visceral fat and metabolic syndrome, which encompasses well recognized risk factors such as obesity and hypertension, and (2) its effects through paracrine and endocrine activity, wherein epicardial fat releases bioactive molecules that influence cardiac morphology and function both positively such as adiponectin, adrenomedullin and omentin and negatively such as interleukin 1 (IL-1), interleukin 6 (IL-6), and tumor necrosis factor (TNF)-alpha [9].

The main inflammatory mediators released by epicardial fat are IL-1, IL-6, interleukin 8 (IL-8), and TNF-alpha. These initiate chronic inflammations, especially in obese individuals [10]. The cytokine IL-6 promotes the proliferation of smooth muscles in vascular walls, which is an essential aspect in the formation of atherosclerotic plaque and endothelial dysfunction [11]. Additionally, it suppresses the expression of the adiponectin gene, an anti-inflammatory hormone, which leads to worsening of hypertension and obesity. It was also found that IL-6 promotes insulin resistance, stimulates gluconeogenesis, and hepatic secretion of triglycerides [12]. TNF-alpha is another important proinflammatory cytokine secreted by epicardial fat. It causes vasoconstriction directly as well as indirectly through increased production of angiotensin II and endothelin-1 [13]. Furthermore, TNF-alpha contributes to the maintenance of chronic inflammation by reducing the release of adiponectin and increasing the synthesis of other proinflammatory chemicals, such as IL-6 [14].

Various studies link the negative role of increased epicardial fat thickness on cardiac function and the severity of CAD. Our study aimed to correlate echocardiographic EFT with CAD severity, examine how risk factors like smoking, diabetes, and obesity impact this relationship and assess EFT's predictive value for CAD severity.

In our study, CAD patients had a mean EFT of 5.24 mm (SD 1.58 mm), which was significantly higher than in non-CAD patients (2.94 mm with SD of 0.89 mm). This highlights a potential link between increased epicardial fat and the occurrence of coronary artery disease, consistent with the findings of Ghaderi et al., who reported a positive association between elevated epicardial fat thickness in CAD compared with non-CAD (3.0±3.69 vs 1.2±3.6, p < 0.0001) [15]. Similarly, Meenakshi et al. found a significant association between increased EFT and CAD, with EFT in CAD patients averaging 6.9 mm (SD 1.9 mm) compared to 4.4 mm (SD 1.2 mm) in non-CAD patients [16].

A strong positive association was also found between EFT and Gensini scores in our study, with Pearson and Spearman coefficients of 0.697 and 0.673, respectively. This aligns with findings from a study done by Sinha et al., where a strong positive association was found between EFT and Gensini score, highlighting EFT's potential as a non-invasive biomarker for assessing CAD risk and severity [17]. Our ROC curve analysis further supports EFT's diagnostic accuracy in predicting CAD severity, consistent with results obtained by Okada et al. in their study [18].

Our findings indicated that the presence of diabetes is significantly associated with increased EFT (4.91±1.86 millimeters vs. 4.03±1.58 millimeters, p = 0.01). According to a meta-analysis of several studies on EFT in diabetic patients conducted by Li et al., it was concluded that diabetics have substantially greater EFT levels than non-diabetics [19].

Our study did not show any significant association between risk factors like alcohol use, hypertension, dyslipidemia, and smoking with EFT. Kazibwe et al. studied the relation of alcohol consumption with ectopic fat deposition and found a J-shaped association wherein heavy alcohol intake and binge drinking led to higher ectopic fat deposition. They found that pericardial fat had the strongest association with alcohol use among the various ectopic fats that they examined [20]. Guan et al. conducted a meta-analysis of multiple studies on EFT in hypertensive patients, and it was concluded that hypertensive patients tend to present with higher EFT thickness and increased EFT might be associated with high risk of non-dipping blood pressure [21]. A study by Monti et al. found that smoking is an important determinant of increased epicardial fat [22]. Donmez et al. conducted a study that concluded that low-density lipoprotein (LDL)-C levels significantly correlated with increased EFT [23].

Various limitations of this study warrant consideration. Ours was a single-center hospital-based study. Most of our patients belonged to the lower or middle socio-economic strata. We had a relatively small sample size. Extrapolating our findings and applying them to the general population at large requires further studies with larger sample sizes and diverse population groups.

## Conclusions

This study provides strong evidence that EFT correlates significantly with the severity of CAD. Higher EFT values are linked to increased CAD severity, as indicated by elevated Gensini scores, suggesting that EFT can serve as a non-invasive biomarker for assessing CAD severity. The presence of diabetes also correlated with increased EFT, highlighting the importance of managing this condition to reduce cardiovascular risk. Further research is necessary to build up on these findings.
